# Innate Immune Programing by Endotoxin and Its Pathological Consequences

**DOI:** 10.3389/fimmu.2014.00680

**Published:** 2015-01-06

**Authors:** Matthew C. Morris, Elizabeth A. Gilliam, Liwu Li

**Affiliations:** ^1^Department of Biological Sciences, Virginia Polytechnic Institute and State University, Blacksburg, VA, USA; ^2^Virginia Tech Carillion School of Medicine and Research Institute, Roanoke, VA, USA

**Keywords:** innate programing, endotoxin, priming and tolerance, systems dynamics, acute and chronic inflammation

## Abstract

Monocytes and macrophages play pivotal roles in inflammation and homeostasis. Recent studies suggest that dynamic programing of macrophages and monocytes may give rise to distinct “memory” states. Lipopolysaccharide (LPS), a classical pattern recognition molecule, dynamically programs innate immune responses. Emerging studies have revealed complex dynamics of cellular responses to LPS, with high doses causing acute, resolving inflammation, while lower doses are associated with low-grade and chronic non-resolving inflammation. These phenomena hint at dynamic complexities of intra-cellular signaling circuits downstream of the Toll-like receptor 4 (TLR4). In this review, we examine pathological effects of varying LPS doses with respect to the dynamics of innate immune responses and key molecular regulatory circuits responsible for these effects.

## Current Dogma and Limitations with Regard to LPS Signaling in Innate Immunity

Lipopolysaccharide (LPS) is a ubiquitous molecule found on the surface of Gram-negative bacteria and is recognized by innate immune cells in humans. Slightly elevated levels of LPS persist in humans with chronic diseases and lifestyles that involve chronic smoking and drinking (1–7). Through a better understanding of how inflammation plays a role in the development of chronic disease, it is possible to devise better treatments to prevent or mitigate their debilitating effects. Currently, it is believed that low-grade inflammation plays a significant role in slowing and preventing normal healing processes from occurring, leading to chronic diseases including heart disease, diabetes, reduced wound healing, and even Parkinson’s disease and rheumatoid arthritis (RA) (2, 8–13).

Lipopolysaccharide challenge is known to induce a refractory state in cells, whereupon subsequent challenge, even with a high dose of LPS, is characterized by less robust induction of pro-inflammatory cytokines and increased production of anti-inflammatory cytokines, a state known as endotoxin tolerance (14–16). The duration of exposure has also been implicated in different immune responses (16). Pretreatment with a very low dose of endotoxin (in the picograms/milliliter range), in contrast, has an opposite effect, potentiating or “priming” the pro-inflammatory response to subsequent endotoxin challenge. This phenomenon is referred to as the Shwartzman-like reaction (17). We and others have documented the priming response to very low-dose LPS *in vitro*, where it results in augmented expression of pro-inflammatory cytokines such as IL-6 and tumor necrosis factor α (TNFα), and *in vivo*, where mice pretreated with super-low-dose LPS exhibit increased mortality in response to challenge with a higher dose (18, 19). Endotoxin priming and tolerance have both been well documented, though the molecular mechanisms governing the decision between one response and the other have not been well defined. Regardless, the “decision” must be made at the time of the primary challenge: since the secondary stimulus can be delivered at precisely the same dosage and for the same duration, the differences in the response cannot originate with the secondary challenge. The difference between priming and tolerance must therefore be in the response to the preparatory dose, and it is here that a detailed examination of the dynamics of the macrophage response to LPS would be most fruitful.

The first events in the immune response to LPS occur outside of the cell. LPS must first come into contact with the LPS-binding protein (LBP). The LPS–LBP complex can then be recognized by TLR4, acting in conjunction with MD-2 and cluster of differentiation 14 (CD14) (20). Once this recognition has occurred, the TLR4 signaling cascade can commence.

**Figure 1:**
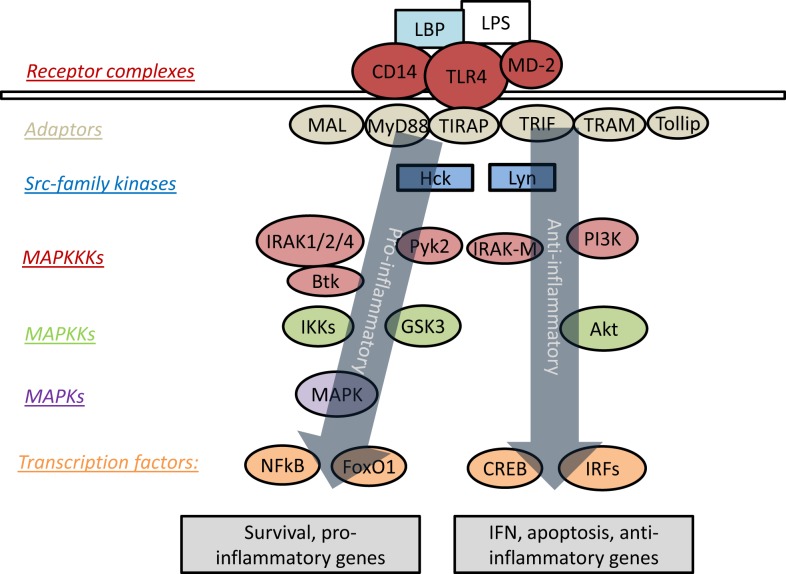
**Overview of signaling cascades engaged by TLR4**. LPS triggers recruitment of various adaptor molecules that may recruit a myriad of downstream kinases and transcription factors, resulting in the activation of both pro- and anti-inflammatory responses. However, the dynamic modulation and coordination of these complex events are not well understood.

Upon ligation of TLR4 by LPS, signaling can proceed through an MyD88-dependent or MyD88-independent pathway. The intra-cellular portion of TLR4 contains a Toll/IL-1R homology (TIR) domain, by which it is enabled to interact with a family of related proteins and adaptor molecules, most prominently MyD88 and TIR-domain-containing adaptor protein inducing interferon-β (TRIF) (21). TLR4 is unique for its ability to signal through both MyD88 and TRIF, as the other TLRs are limited exclusively to either MyD88-dependent or TRIF-dependent signaling (22). Recruitment of MyD88 to TLR4 is followed by a signaling cascade involving the interleukin-1-receptor-associated kinases or interleukin-receptor-associated kinase (IRAKs). There are currently four known IRAKs, among which IRAK-1, -2, and -4 play positive roles in signal transduction, while IRAK-M (also known as IRAK-3) acts to suppress TLR signaling (23). The MyD88-dependent pathway of TLR signaling culminates in the activation of mitogen-activated protein kinases (MAPK) and NFκB, with subsequent induction of pro-inflammatory genes (24). Figure 1 presents an overview of prominent mediators of TLR4 signaling.

Signaling through TRIF usually requires endocytosis of TLR4 (25). This endocytosis in turn requires CD14, as interference with CD14–TLR4–LBP interactions prevents effective TLR4 internalization (26, 27). Interestingly, Watanabe et al. demonstrated that TRIF-dependent signaling can be activated in the absence of CD14 (28), but this required the direct delivery of LPS to the interior of the cell, suggesting even more strongly that TRIF-dependent signaling requires signaling by TLR4 within the confines of the cytoplasmic membrane. A single amino acid mutation in TLR3 is sufficient to induce signaling through MyD88, rather than TRIF, indicating that the two pathways are closely related (29). Once recruited, TRIF in turn activates interferon regulatory factor 3 through TBK1, and signaling proceeds through PI3K, ultimately resulting in the activation of interferon-β (IFNβ) and related genes (14). LPS preconditioning has TRIF-dependent protective effects with respect to the ischemic injury associated with stroke (30), and deletion of TRIF exacerbates allergic dermatitis in mice (31). Taken together, these results point to a broadly anti-inflammatory role for TRIF-dependent signaling in addition to its established pro-inflammatory effects downstream of TLR4 activation.

## Emerging Concept of Innate Programing and Memory

Past studies have largely treated the MyD88-dependent and TRIF-dependent pathways in isolation, with little attention paid to the possibility of cross-talk between them. However, there are intriguing indications that such cross-talk does occur. IRAK-1, a pivotal actor in MyD88-dependent signaling, plays a suppressive role in TRIF-dependent TLR3 signaling (32), and MyD88 is important for the suppression of TRIF-mediated apoptosis (33). The Src-family tyrosine kinases (SFK) also play a role in the differential regulation of pro- and anti-inflammatory effects downstream of TLR stimulation (34), including direct involvement in MyD88-dependent NFκB activation (35). The phenomena of endotoxin priming and tolerance indicate that the pro- and anti-inflammatory responses to TLR4 stimulation are not wholly independent; rather, the activation of one must affect the other somehow. These emerging studies hint at a novel concept of “innate immune programing” and “memory.” Based on the mutually inhibitory cross-talks among these pathways, innate leukocytes may be skewed to distinct phenotypes and retain certain memory states, such as M1, M2, and other intermediate states (36). However, the mechanisms responsible for this potential memory are not well understood. Our future efforts will be dedicated to the review of potential leads that may help reveal the underlying mechanisms.

The major mediators of cross-talk between MyD88- and TRIF-dependent TLR4 signaling appear to be the SFKs and phosphatidylinositol-3-kinase (PI3K), which act at different “layers” downstream of the receptors to integrate signals from the different pathways. The SFKs are engaged by tumor-necrosis-factor-receptor-associated factor 6 (TRAF6) (37), and activated within minutes of LPS stimulation, along with the Syk kinase Pyk2 and Bruton’s tyrosine kinase (Btk), a member of the Tec family (38). The SFK Lyn has been studied chiefly in B cells, due to the spontaneous appearance of a lupus-like B-cell-mediated autoimmune disease in Lyn-deficient mice (39). Lyn activates PI3K through B-cell adaptor for PI3K (BCAP) in B cells (40), but DC-specific deletion of Lyn still causes hyperactive MyD88 signaling and B cell-mediated autoimmunity (41), pointing to a role for Lyn in myeloid cells. Knockout of MyD88 either globally or conditionally in B cells or dendritic cells counteracts the autoimmune symptoms characteristic of Lyn deficiency (42, 43). BCAP itself is active in myeloid cells as well (44), indicating that the network is not limited to lymphoid cells. In mast cells, Lyn is necessary for TLR4-dependent NFκB and MAPK activation (45), and further contributes to the activation of Btk (46), a TIR-domain-containing molecule, which promotes LPS-induced NFκB activation in macrophages (47). Btk also drives MAPK-dependent TNFα production in response to TLR2 and TLR4 stimulation of myeloid cells (48).

Inhibition of Pyk2 ameliorates the symptoms of LPS-induced lung injury (49), and it also promotes MyD88-dependent signaling and NFκB activation (35). Together, these findings point toward a generally pro-inflammatory role for Pyk2, but the discovery that PI3K inhibitors suppress Pyk2 activity (50) indicates that Pyk2 is involved in both pathways. Downstream of PI3K, Pyk2 may act in part to modulate the inflammatory suppression driven by PI3K/Akt signaling.

The need for further study of the regulation of pro- and anti-inflammatory responses to TLR4 stimulation is clear. Since the “switch” between endotoxin priming and tolerance appears to depend on the dosage of the first challenge, the dynamics of that response must be investigated with the aim of determining what conditions lead to the activation of one pathway or another. The network motifs active here are key to the understanding of the LPS response. There exists a role for epigenetics in the broad reprograming of macrophages and endotoxin tolerance (51–53), but the response to single dosages over a matter of hours is likely to be regulated by faster dynamic molecular mechanisms, as discussed below in further detail.

## Competitive Circuitry Governing Innate Programing of Leukocytes by LPS

A growing body of literature suggests that a competitive network may be responsible for the decision between a predominantly pro- or anti-inflammatory response to LPS, with PI3K, Akt, and the cAMP-response-element-binding-protein (CREB) acting in opposition to glycogen synthase kinase 3 (GSK3) and forkhead box O1 (FoxO1). BCAP, as mentioned above, is crucial for TLR-dependent PI3K activation in myeloid cells and the ensuing suppression of inflammation (44). PI3K dampens NFκB activation by means of phosphoinositide-dependent kinase-1, which suppresses TRAF6 activity and is necessary for the LPS-induced activation of Akt and ERK (54). PI3K also activates Akt in response to mammalian target of rapamycin (mTOR), competing with MAPK/p38/JNK signaling (55). Inhibition of PI3K leads to increased production of IL-6 and TNFα in response to TLR2 stimulation of macrophages (56), and activation of PI3K results in deactivation of FoxO1, preventing it from promoting TLR4 signaling (57). Overall, PI3K is important for negative feedback and control of TLR signaling, acting to counteract both NFκB and MAPK, two of the main transcription factors responsible for pro-inflammatory gene transcription in response to LPS.

Akt exerts its anti-inflammatory effects through NFκB and MAPK signaling, as well as its activation of CREB. In non-canonical NFκB signaling, the processing of p100–p52 requires Akt, paving the way for increased activity of RelB (58), a suppressive NFκB family member. Activation of Akt through the mTOR–PI3K pathway both counteracts MAPK signaling and activates CREB (59), at the same time directly inactivating FoxO1 (55). Quercetin treatment activates Akt in multiple cell types, leading to decreased activity of FoxO1 in pancreatic islets (60), and ameliorating the inflammatory response of adipocytes to TNFα (61). Akt activation has also been shown to correlate with suppression of FoxO1 in HEK293 cells (62). Inhibition of JAK3 leads to decreased activity of both Akt and CREB, and this loss of activity correlates with an augmented pro-inflammatory response to LPS (63). The role of Akt, then, seems to be to mediate the anti-inflammatory effects of PI3K, in large part by suppressing FoxO1 and activating CREB.

GSK3 has been implicated in many inflammatory signaling pathways. It directly suppresses genes with CREB binding sites (64). Inhibition of GSK3 increases IL-10 production and decreases IL-12 in response to LPS in monocytes (65), which, in light of the importance of IL-10 to the anti-inflammatory effects of PI3K (66), points strongly to GSK3 as an actor in opposition to PI3K. GSK3 suppresses IFNβ induction by LPS (67), indicating a suppressive effect on TRIF-dependent signaling. GSK3 inactivates CREB directly (68, 69), and IFNγ activates GSK3 and suppresses CREB (70), indicating that the pro-inflammatory effects of IFNγ may be due in large part to its effects on this sub-network.

**Figure 2:**
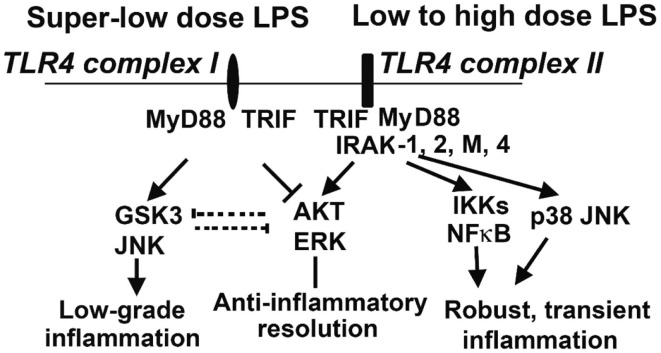
**Competitive molecular circuits potentially responsible for the dynamic programing of innate leukocytes**. Varying doses of LPS may engage unique TLR4 complexes that may cause intra-cellular pathway switching, leading to either low-grade non-resolving inflammation, or acute resolving inflammation. Super-low-dose LPS selectively activates GSK3 and JNK, while suppressing AKT and ERK. These two branches may also have mutually inhibitory interactions, further fine-tuning cellular inflammatory states. Low- to high-dose LPS potently induces NFκB and MAP kinases, leading to robust inflammatory reactions. On the other hand, low- to high-dose LPS could also trigger the activation of AKT and ERK, which may serve to dampen inflammatory responses. IRAK-1 appears to be critical for both the low-grade inflammation triggered by super-low-dose LPS and the anti-inflammatory responses (e.g., IL-10 expression through ERK activation) activated by high-dose LPS.

The opposing effects of PI3K activation and GSK3 activation have been described in multiple cell types. They have opposing effects on the LPS response in DC (66), and in H_2_O_2_-induced apoptosis in neurons (71). Inactivation of GSK3β is important in inflammatory resolution and is associated with a blunted pro-inflammatory response to LPS (72). In macrophages, PI3K–Akt signaling directly opposes GSK3 activity during the LPS response, with GSK3α knockdown potentiating the effects of IL-10 while CREB knockdown reduces them. Furthermore, the pro-inflammatory effects of PI3K inhibition can be counteracted by treatment with IL-10 (73), another indicator that IL-10 is a downstream effector of PI3K. The PI3K-dependent increase in IL-10 production is due to its inactivation of GSK3 (67, 74), and direct activation of PI3K–Akt results in inhibitory phosphorylation of GSK3 (75). Knockout of the mTOR signaling molecule rictor prevents Akt from inactivating GSK3 upon TLR4 stimulation, correlating with increased FoxO1 activity and pro-inflammatory gene expression (76). Taken together, these findings constitute a strong body of evidence that the anti-inflammatory PI3K/Akt/CREB signaling axis acts by suppressing GSK3/FoxO1, and that this competition is the lynchpin of the primarily pro- or anti-inflammatory characteristics of the dynamic LPS response (Figure 2). We recently reported that super-low-dose LPS selectively activates GSK3 and JNK while suppressing Akt and ERK (77). This may explain the mild skewing of pro-inflammatory responses by low-grade endotoxemia in mice and humans. In contrast, high-dose LPS can induce robust activation of all MAPKs that include p38, JNK, and ERK, as well as PI3K/Akt (77), which may lead to the robust yet transient resolving inflammation followed by anti-inflammatory tolerance associated with high-dose endotoxin challenge. With regard to upstream signaling network, IRAK-1 is responsible for the effects of super-low-dose and high-dose LPS (Figure 2) (77, 78).

## Pathological Effects of Varying Dosages of Endotoxin

Chronic diseases currently affect large proportions of the US population where currently one in three adults is obese and almost one in five children between 6 and 19 years are also considered obese (79–82). In 2005, the CDC estimated that one in two Americans suffered from at least one chronic disease, such as arthritis, greatly decreasing their quality of life and participation in daily activities (83).

Lipopolysaccharide is a ubiquitous molecule found on the surface of Gram-negative bacteria and is recognized by innate immune cells in humans. Slightly elevated levels of LPS persist in humans with chronic diseases and lifestyles that involve chronic smoking and drinking (1–7). Elevated circulating endotoxin may program host leukocytes into a low-grade “memory” state, and contribute to the pathogenesis of diverse diseases that may include atherosclerosis, diabetes, reduced wound healing, Parkinson’s disease, and RA (2, 8–13). Indeed, recent studies suggest that the lower circulating levels of endotoxin may lead to enhanced pathogenesis of atherosclerosis (84, 85). Elderly people tend to have elevated circulating endotoxin associated with neurological disease (86, 87). On the other hand, slightly elevated endotoxin *in vivo* may offer protection toward ischemia reperfusion injuries (88–91). Varying dosages of endotoxin may also affect the function of dendritic cells, and alter vaccine efficacies (92).

**Figure 3:**
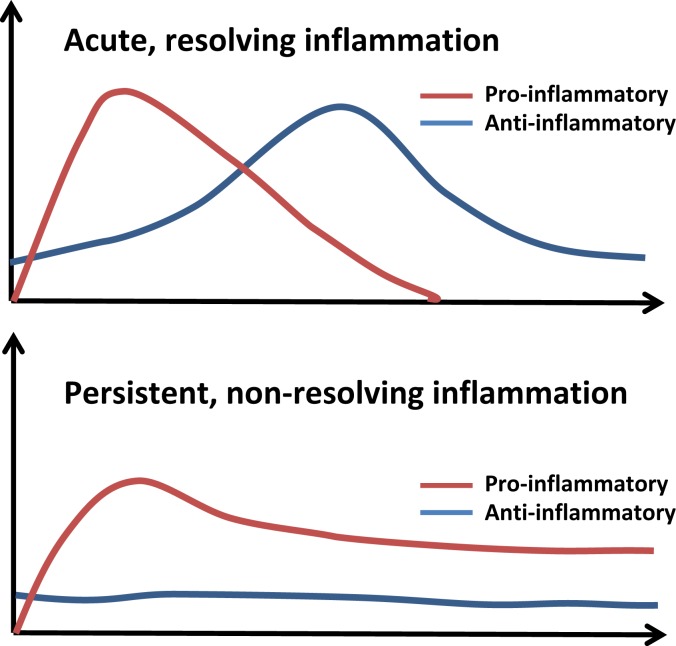
**The course of acute and persistent inflammatory responses**. The upper panel depicts the trajectory of a typical inflammatory response. An initial pro-inflammatory phase characterized by recruitment of neutrophils and production of cytokines such as IL-6 and TNFα is followed by resolution, with compensatory induction of anti-inflammatory cytokines (e.g., IL-10, TGFβ). Non-resolving and persistent inflammation is mild but unopposed by anti-inflammatory mechanisms.

To put these dynamic pathological and physiological responses in perspective, we have simplified these contrasting profiles of acute and persistent inflammation in Figure 3. A normal inflammatory response comprises an early, pro-inflammatory phase, in which microbicidal functions predominate, and a secondary, anti-inflammatory phase, where wound healing occurs and inflammatory cells leave the area of damage (72, 93, 94). In chronic disease, the pro-inflammatory phase fails to resolve, leading to a persistent state of low-grade inflammation. This leads to changes in mucosal barriers and commensal bacteria that line the gastrointestinal tract. As a result, these individuals tend to have slightly elevated levels of LPS (1–100 pg/ml) circulating in their blood (1, 2, 95–100). However, while inflammatory processes for high doses of LPS (>10 ng/ml) have been intensively studied for its role in septic shock (101, 102), much less is known about the immunological response to subclinical doses of LPS. Typically, the activation of the Toll-like receptor-4 (TLR4) complex leads to the activation of nuclear factor κB (NFκB) where it initiates the transcription of genes encoding inflammatory cytokines (103–108). These inflammatory cytokines are responsible for the recruitment of neutrophils, natural killer cells, and antigen-presenting cells to the site of the infection (103). Once the infection is cleared, other cytokines such as IL-10 and transforming growth factor β (TGFβ) combine with apoptosis of pro-inflammatory cells such as neutrophils to resolve inflammation and restore homeostasis (103). Additionally, macrophages can enter a state of endotoxin tolerance wherein they suppress their expression of pro-inflammatory mediators to prevent excessive inflammation (109, 110). Suppression at multiple levels including inhibitor of κB, phosphatidylinositol-3-kinase (PI3K), MAP kinase phosphatases, and the inactivation of IRAK-1 helps prevent inflammation in the absence of danger signals (54, 56, 111).

It is important to note that “innate programing” and “memory” may not be limited exclusively to endotoxin responses. Rather, recent studies have indicated that other microbial products such as beta-glucan can analogously “train” or program host innate immunity, and led to improved host responses toward controlling infection (112–114).

## Conclusion

Remaining questions include the plasticity of this network, and the persistence of the programing for which it is responsible. Can the character of the LPS response be easily altered by pharmacological intervention aimed at activating or inhibiting different components of this signaling nexus? Once the character of the response has been established, will the system reset itself? Answers to these questions will significantly advance the understanding of TLR signaling in particular and the behavior of innate immune cells in general. Some efforts have been made to apply large-scale systematic methods to the study of this system (115, 116), but a great deal of work remains to be done, particularly with respect to the network herein described. There is a growing appreciation for plasticity and memory in macrophages, with a movement away from strict classifications of macrophage populations along lines of classical/alternative activation to more flexible schemes of classification based on dedication to a variety of different functions (36, 117). It is likely that further examination of this and other myeloid signaling networks will accelerate this. Innate immune “memory” is not a function of dedicated cell types as in lymphoid cells but rather a characteristic intrinsic to individual cells, whereby signals percolating through a network change its state in such a way as to influence its responses to subsequent stimuli. Such “memory” is therefore likely to be an important characteristic of many different cell types, particularly those responding to many different stimuli through interlocking networks of receptors and signaling cascades (neurons, in particular, come readily to mind). Innate immune cell populations may come to be seen as temporary workers, dedicated to their functions less strongly than has hitherto been supposed. Increasing appreciation for this plasticity will open broad new vistas for both the theoretical understanding of innate immunity and the treatment of associated diseases. Further studies aimed at the unique characteristics of innate memory and the underlying mechanisms are urgently needed.

## Conflict of Interest Statement

The authors declare that the research was conducted in the absence of any commercial or financial relationships that could be construed as a potential conflict of interest.

## Abbreviations

BCAP, B-cell adaptor for PI3K; Btk, Bruton’s tyrosine kinase; CD14, cluster of differentiation 14; CREB, cAMP response-element-binding protein; C/EBPδ, CCAAT-enhancer-binding protein δ; EPC, endothelial progenitor cell; FoxO1, forkhead box O1; GBP-1, guanylate-binding protein 1; GSK3, glycogen synthase kinase 3; IFN, interferon; IRAK, interleukin-receptor-associated kinase; LBP, lipid-binding protein; LPS, lipopolysaccharide; MAPK, mitogen-activated protein kinase; mTOR, mammalian target of rapamycin; NFκB, nuclear factor κ of activated B cells; PI3K, phosphatidylinositol-3-kinase; RA, rheumatoid arthritis; SFK, Src-family kinase; TIR, Toll/IL-1R homology; TGFβ, transforming growth factor β; TNFα, tumor necrosis factor α; TRAF6, tumor-necrosis-factor-receptor-associated factor 6; TRIF, TIR-domain-containing adaptor protein inducing interferon-β.
